# Robust multiresonant nonlocal metasurfaces by rational design

**DOI:** 10.1515/nanoph-2024-0551

**Published:** 2025-02-04

**Authors:** Stephanie C. Malek, Chloe F. Doiron, Igal Brener, Alexander Cerjan

**Affiliations:** Center for Integrated Nanotechnologies, 559754 Sandia National Laboratories, Albuquerque, NM, 87185, USA

**Keywords:** metamaterials, metasurfaces, quasi-bound states in the continuum, symmetry breaking

## Abstract

Dielectric metasurfaces supporting optical resonances have become a promising platform for quantum and nonlinear optics. However, resonant metasurfaces remain limited in their capacity to independently control the behavior of many distinct resonances despite efforts in computational optimization and inverse design. In this work, we overcome longstanding limitations by introducing a generalized rational design paradigm based on symmetry. Specifically, we use symmetry-broken metasurfaces with periodic “quadromer” lattices comprised of four nanostructures per unit cell to enable extensive control of multiple optical resonances. The rationally designed metasurfaces are readily fabricable, and we experimentally demonstrate metasurfaces that support up to four high Q-factor resonances with deliberately chosen free-space polarizations, spectral separations, and mode profiles. Our design paradigm may unlock new applications for multiresonant metasurfaces in quantum and nonlinear optics, optical sensing, and augmented reality displays.

## 1 Introduction

Free-space optical metasurfaces offer spatial or spectral control over many fundamental properties of light, such as phase, amplitude, and polarization [1], [2]. While “local” metasurfaces typically spatially shape a wavefront to achieve devices such as metalenses and metasurface holograms, “nonlocal” metasurfaces [3], [4] engineer the optical spectrum via spatially extended optical resonances whose frequency is dependent on the in-plane momentum. In the context of nonlocal metasurfaces, symmetry-protected quasi-Bound States in the Continuum (q-BICs) have proven to be among the most readily engineerable optical resonances due to their generalizable, high-level design rules [5], [6]. Bound States in the Continuum (BICs) are nonradiative modes with infinite optical lifetimes despite existing in the radiation continuum [7], [8]. BICs supported by a periodic lattice can become radiative q-BICs with a periodic symmetry-breaking perturbation [9]. The particular symmetry-breaking perturbation dictates which bound modes become radiative q-BICs and which polarization of free-space light can excite a given q-BIC at normal incidence, and the relationship between metasurface symmetry, q-BIC mode profile, and free-space polarization follow known “selection rules” [6]. Regardless of the type of symmetry-breaking perturbation, the strength or extent of the perturbation (δ) influences the optical lifetime or quality factor (Q-factor) as $Q\propto \frac{1}{\delta^{2}}$ [5], [10]. The simplest manifestation of a metasurface that supports q-BICs is a periodic monomer lattice with one nanostructure per unit cell, designed where selected symmetry operations are broken [9], [11], [12], [13], [14]. A popular and more complex alternative is a periodic dimer lattice with two nanostructures per unit cell [15], [16], [17], [18], [19], [20]. Relative to monomer lattices, dimer lattices support additional q-BICs controlled with more specificity via a comparatively larger selection of symmetry operations [6].

Unfortunately, a lingering fundamental challenge in nonlocal metasurfaces has impeded their prospects for technological maturation: Nonlocal metasurfaces typically struggle to exert sufficient control over the behavior and spectral separation of multiple optical resonances simultaneously. Approaches to doubly resonant metasurfaces have included inverse design [21], coupling a metasurface to an external optical cavity [22], or combining distinct metasurfaces such that different patches of the device have distinct resonant frequencies [23]. A few nonlocal metasurfaces have experimentally demonstrated systematic control over two q-BICs through symmetry [24], [25]. However, existing doubly resonant designs are not readily scalable to supporting several resonances such that each resonance has deliberately selected properties. Multiresonant metasurface designs are often incompatible with ordinary nanofabrication limitations and imperfections, resulting in multiresonant metasurfaces that have either not been demonstrated experimentally [26] or show significantly worse experimental performance than expected [27]. Even though dimerized nonlocal metasurfaces typically excel at controlling q-BICs, they are unsuitable for highly multiresonant metasurfaces because they have neither sufficient symmetry operations to independently control the Q-factor and polarization of more than two or three q-BICs [6] nor enough geometrical parameters to reliably control the spectral spacing between q-BICs [28].

In this work, we propose and experimentally demonstrate a rational design scheme based on symmetry to simultaneously engineer the optical lifetimes, free-space polarizations, mode profiles, and resonant wavelengths of up to four distinct optical resonances. To do so, we investigate “quadromer” lattices with four nanostructures per primitive unit cell. Quadromer lattices manifest an unusual physical phenomenon of not just “folding” the First Brillouin Zone but instead folding it twice, which introduces sets of BICs and q-BICs that are not accessible together in conventional lattices. We then use group theory to develop intuitive heuristics for the relationships between quadromer lattice symmetry, whether each band-folded mode is a BIC or q-BIC, and, if applicable, the free-space polarization of each q-BIC. As a proof of concept, we experimentally demonstrate optical behaviors that are not achievable in dimer lattices, such as well-controlled pairs of copolarized q-BICs and a copolarized set of four q-BICs from silicon quadromer metasurfaces operating at near-infrared wavelengths. The capability to not only design and but also faithfully fabricate multiresonant metasurfaces should enable novel metasurfaces that produce complex entangled photon states [29], [30], generate nonlinear optical signals [21], [28], [31] with competitive efficiency, identify complex molecules with improved accuracy [32], [33], and reduce the form factor of multicolor optical combiners in augmented reality headsets [24], [34], [35].

## 2 Results

**Figure 1:**
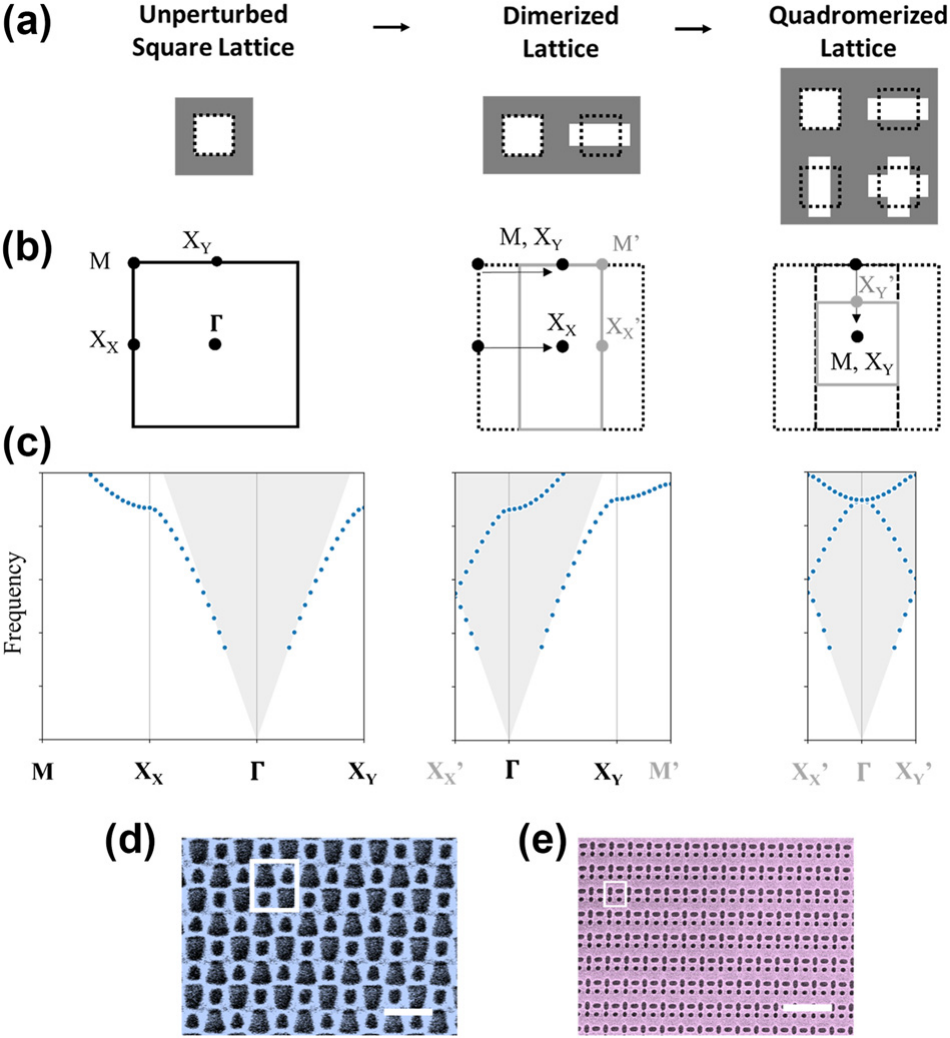
Conceptualization of periodic quadromer lattices. (a–c) Formulation of quadromer lattices by two successive period doubling perturbations corresponding to two successive band folding operations. (a and b) Schematics of monomer, dimer, and quadromer in (a) real-space (primitive unit cell) and (b) *k*-space (First Brillouin Zone). (c) Band diagram (fundamental band only) for monomer, dimer, and quadromer. (d and e) False color scanning electron micrographs of quadromerized metasurfaces fabricated in silicon on a glass substrate. Scale bars: 1 µm. White box indicates one primitive unit cell.

To develop multiresonant nonlocal metasurfaces, we consider the photonic bandstructures of doubly dimerized or “quadromerized” lattices with four nanostructures per unit cell. Figure 1a and b (left column) shows a subwavelength periodic square lattice and its photonic bands (Figure 1c, left column); X_X_ and X_Y_ are equivalent high symmetry points in an unperturbed square lattice [36]. Adding a dimerizing perturbation along the *x*-direction to double the period in real space (Figure 1a, middle column) also halves the period in *k*-space. This folds the photonic band structure (Figure 1b and c, middle column) so that the **X**_**X**_ (**M**) point in the monomer lattice moves to **Γ** (**X**_**Y**_) in the dimerized lattice. We repeat the same procedure in the *y*-direction to form a quadromer lattice with four nanostructures per unit cell (Figure 1a–c, right column). The second dimerization folds both **M** and **X**_**Y**_ to **Γ**. As a result, the **Γ**-point of the quadromer lattice has two copies of **X**-point modes that were equivalent in the unperturbed lattice [6], which makes quadromer lattices particularly well-suited to realizing pairs of BICs or q-BICs where both modes belong to the same photonic band. The symmetry of the quadromer lattice dictates whether each of the modes folded to **Γ** remains a BIC or becomes a radiative q-BIC that is excitable from free-space at normal incidence. There are numerous possible variations of rectangular quadromer lattices with many options for their optical behavior. Two examples of fabricated quadromer lattices are shown in Figure 1d and e.

**Figure 2:**
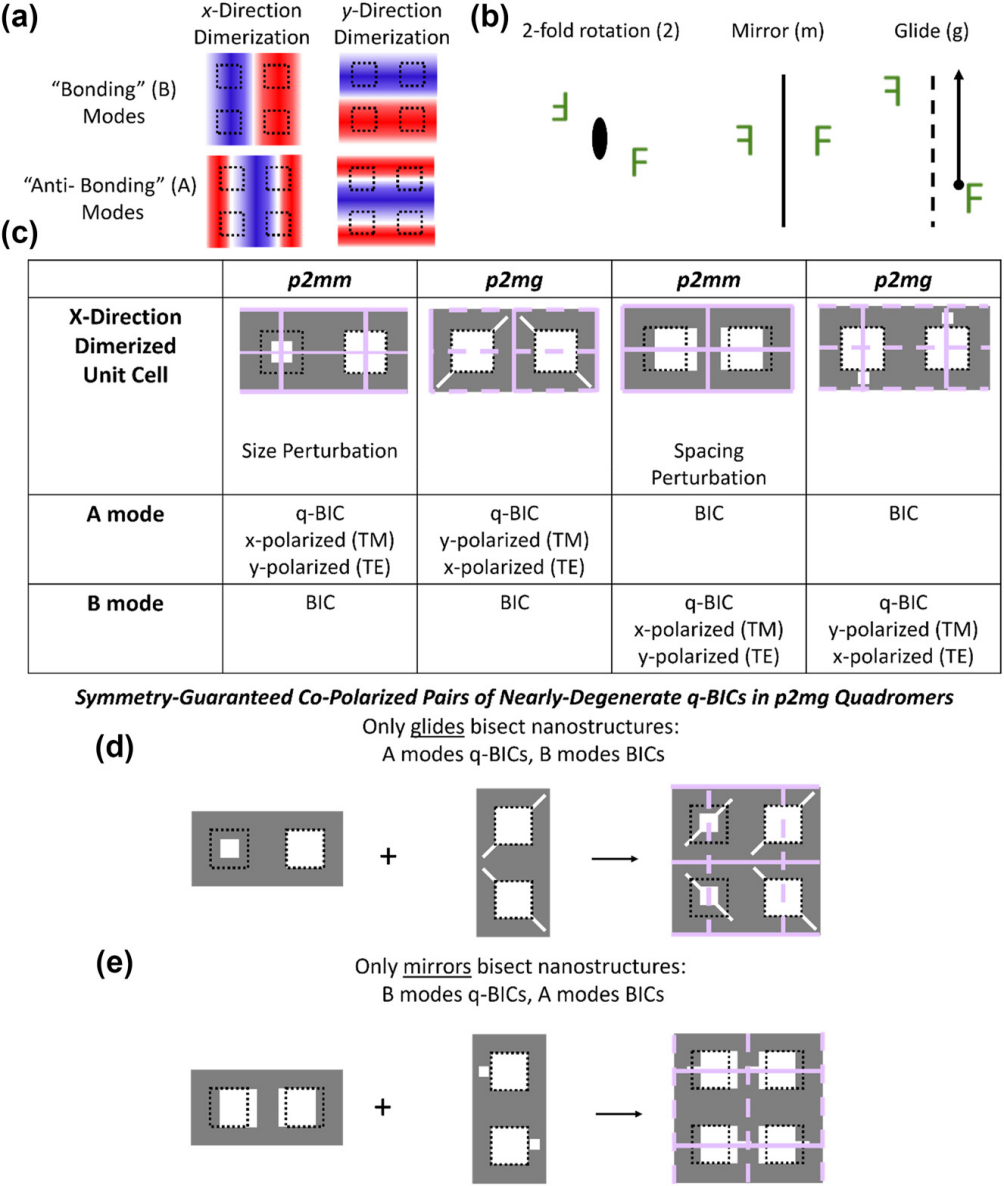
Construction of copolarized pairs of q-BICs. (a) Field profiles for selected modes in quadromerized lattices. (b) Relevant symmetry operations. (c) Review of selection rules for the fundamental dimerized lattices that fold in modes from one **X**-point. TE indicates dominant field component in propagation direction is magnetic field. Selected symmetry operations are indicated on lattice schematics: Mirrors are denoted by solid lines and glides with dashed lines. (d and e) Formulation of *p*2*mg* quadromer lattices supporting copolarized pairs of (d) antibonding and (e) bonding q-BICs by combining selected lattices.

We construct quadromer lattices from dimer lattices, so it is necessary to review the relevant physics of dimer lattices. For simplicity, we consider only a subset of all the possible modes [6], [36]: The “antibonding” (A) and “bonding” (B) modes folded to **Γ** from **X**_**X**_ and **X**_**Y**_ (Figure 2a, field profiles are the out-of-plane component [36] of either the electric (TM) or magnetic (TE) field). The modes folded from the **X**_**X**_ point are rotated 90° relative to those folded from the **X**_**Y**_ point, and both modes of the same type (i.e., A or B) originate from the same photonic band. Quadromerizing a lattice folds all four selected modes to Γ, but the folded modes can be either BICs or radiative q-BICs. For a mode at Γ to be a radiative q-BIC, the perturbation must induce an effective net in-plane dipole moment to the out-of-plane component of either the electric (TM modes) or magnetic (TE modes) field of the mode [6], [10]. Whether specific modes are q-BICs or BICs can be predicted in a generalized way from the symmetries of the lattice and the mode [6]. We, therefore, classify lattices by their symmetry, and relevant symmetry operations are shown in Figure 2b. An important symmetry operation that is atypical in photonic structures is the glide symmetry operation where a lattice is invariant under a mirror plus a translation by half of a lattice vector parallel to the mirror. Figure 2c shows the fundamental dimer lattices that fold **X**_**X**_ to **Γ**, along with their known selection rules [6] for A and B q-BICs and BICs. The selection rules are applicable to metasurfaces made of pillars or holes [6]. We denote these lattices as fundamental lattices because all other *x*-dimerized lattices can be formed from combinations of the fundamental lattices to inherit their q-BICs and shared symmetry operations [6], [26]. Of the fundamental lattices, two belong to the crystallographic wallpaper group *p*2*mm* with perpendicular mirrors while the other two lattices are *p*2*mg* with perpendicular glides and mirrors. Each lattice supports either an A or B q-BIC (and a B or A BIC), and its selection rules are determined by the lattice symmetry and which symmetry operations intersect the nanostructures.

We focus on quadromer lattices that enable the novel capability of copolarized pairs of q-BICs where both q-BICs have the same free-space polarization. Monomer and dimer lattices only support cross-polarized pairs of q-BICs, i.e., where the q-BICs correspond to orthogonal free-space polarizations [6]. Quadromer lattices supporting copolarized pairs of q-BICs are realized by combining *x*-direction and *y*-direction fundamental dimer lattices, but only particular combinations of lattices support copolarized q-BICs. For example, applying the same dimerizing symmetry perturbation in both the *x*- and *y*-direction, or equivalently combining an *x*-dimerized lattice with a 90° rotated copy of itself, generates a quadromer that supports a pair of q-BICs. The q-BICs are cross-polarized because rotating the lattice by 90° also rotates the allowed free-space polarization by 90°, and the lattice has either orthogonal mirrors (*p*2*mm*) or orthogonal glides (*p*2*gg*) (Fig. S1). It follows that copolarized pairs of q-BICs require distinct symmetry perturbations in the *x*- and *y*-directions, which can be achieved by combining particular *p*2*mm* and *p*2*mg* lattices such that the resulting quadromer is a *p*2*mg* lattice with intersecting mirrors and glides (Figure 2d and e). For example, combining an *x*-dimerized *p*2*mm* lattice that supports an A q-BIC (folded to **Γ** from **X**_**X**_) with a *y*-dimerized *p*2*mg* lattice that also supports an A q-BIC (folded from **X**_**Y**_) results in a *p*2*mg* quadromer where glides but not mirrors bisect the nanostructures (Figure 2d). The same logic applies to forming *p*2*mg* quadromers from dimerized lattices that support B q-BICs but mirrors, not glides, bisect the nanostructures (Figure 2e). A rigorous derivation of such behavior through group theory is shown in Supplementary information Section 2. We emphasize that the q-BICs in each pair were equivalent in the unperturbed lattice, have the same mode profile except for a 90° rotation, and belong to the same photonic band. Notably, as the band undergoes equivalent folding from different directions, the bandstructure of the two q-BICs around **Γ** also approximately become rotated copies of each other (Fig. S4). A *p*2*mg* quadromer supports a mismatched pair of copolarized q-BICs from different bands (e.g., A q-BIC from **X**_**X**_ and B q-BIC from **X**_**Y**_) if neither mirrors nor glides bisect the nanostructures or, alternatively, if both mirrors and glides bisect the nanostructures. In this case, the q-BICs have different mode symmetry and were not degenerate in the unperturbed lattice. While other quadromer lattices with lower symmetry can also support copolarized pairs of q-BICs, only the *p*2*mg* lattices in Figure 2d and e guarantee such behavior as other lattices lack sufficient symmetry operations to determine the selection rules of a quadromer.

**Figure 3:**
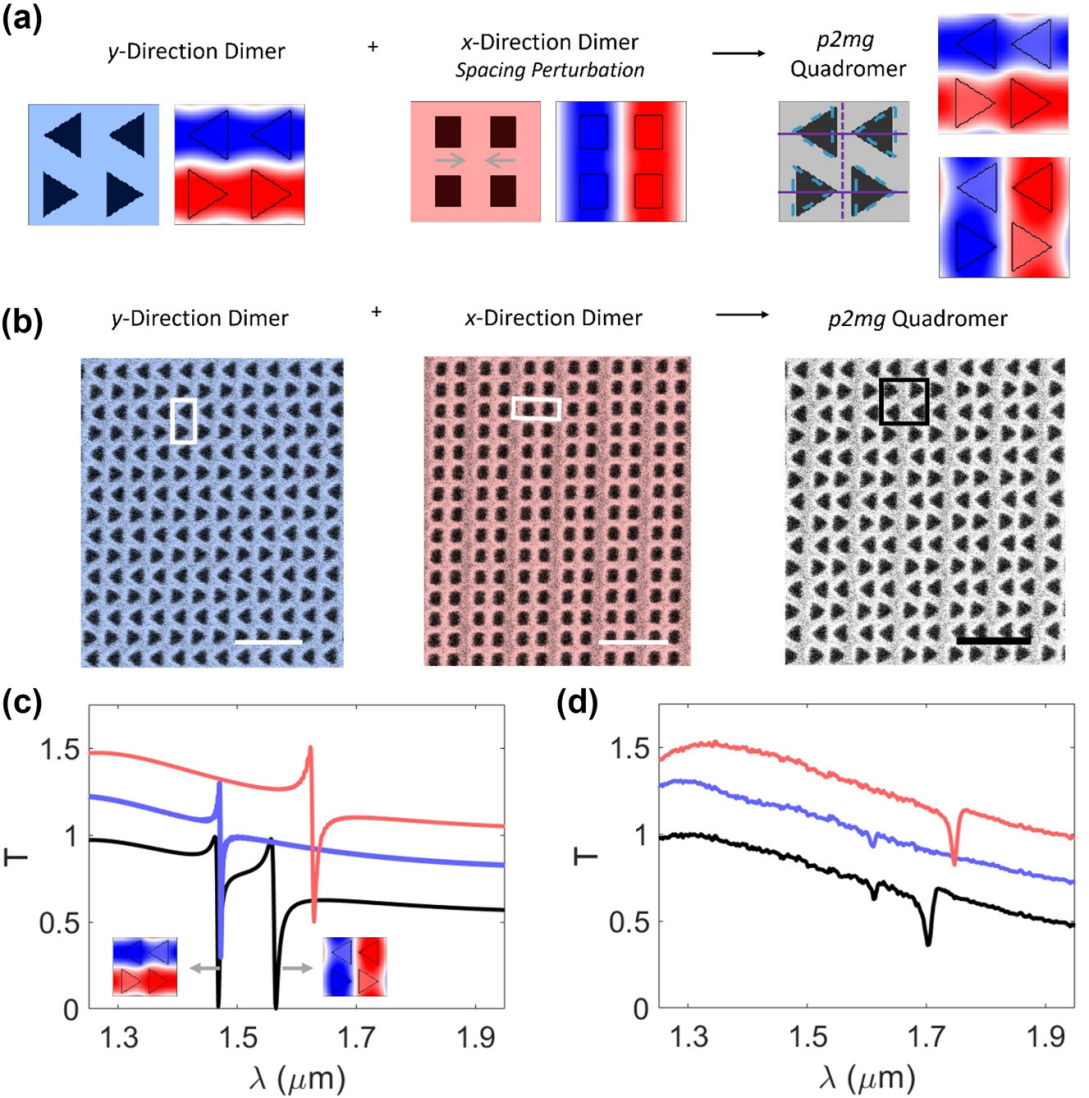
Experimental demonstration of copolarized pairs of q-BICs formulated by combining dimerized lattices. (a) Construction of *p*2*mg* quadromer (with mirrors and glides marked with solid and dashed lines, respectively) supporting a pair of B q-BICs from *x*- and *y*-direction dimers. (b) False color scanning electron microscope images of fabricated metasurfaces. Scale bars: 1 µm. Boxes indicate one primitive unit cell. Device dimensions detailed in Fig. S5. (c and d) Simulated (c) and measured (d) transmission for *y*-polarized incident light. Blue curve: *y*-direction dimer. Red curve: *x*-direction dimer. Black curve: quadromer. Each curve offset by 0.25. Insets in (c): Out-of-plane component of magnetic field of q-BICs.

We experimentally demonstrate a quadromerized nonlocal metasurface with copolarized q-BICs. We first develop a *p*2*mg* quadromer lattice that supports a copolarized pair of B q-BICs by combining a *p*2*mg* lattice dimerized in the *y*-direction with a *p*2*mm* lattice dimerized in the *x*-direction (Figure 3a). The *x*-direction dimerization is a spacing perturbation such that each nanostructure is closer to one of its neighbors. We design and fabricate the metasurfaces as apertures etched into a 200 nm thin film of silicon on a glass substrate. Figure 3b shows scanning electron microscope images of a *p*2*mg* lattice dimerized in the *y*-direction (blue), a *p*2*mm* lattice dimerized in the *x*-direction (red), and the *p*2*mg* quadromer realized by combining the two dimerized lattices (gray). We measure the transmission spectra from each of the three metasurfaces for *y*-polarized (Figure 3d, TE modes) and *x*-polarized (Fig. S5, TM modes) incident light. Our experiments confirm that the spectrum of the quadromer is predicted by the combined spectra of its two constituent dimers, with one q-BIC of the pair contributed by each dimer. We attribute the mild discrepancies between experiments and simulation (Figure 3c) to sidewall roughness, subtle variations in nanostructure sizes, and uncertainties in material refractive indices. Additionally, the measured transmission contrast of each resonance is diminished by the spectral resolution of the spectrometer. As the metasurfaces are nonlocal, the resonant wavelengths of both q-BICs are dispersive with the angle of the incident light, which is relevant because the focused incident beam introduces a slight variation in incident angle (approximately ± 6°) (Fig. S6) and contributes to disparities between measured and simulated spectra. While we have shown metasurfaces of holes etched in silicon, the design rules are generalizable to metasurfaces made of pillars. Figure S7 demonstrates a *p*2*mg* quadromer metasurface made of pillars that supports a pair of copolarized B q-BICs. We introduce quadromer lattices as a combination of *x*-dimerized and *y*-dimerized lattices for simplicity. However, a *p*2*mg* quadromer following the constraints in Figure 2d and e will also support copolarized q-BICs even if one of its dimerizing perturbations is along the 45° diagonal direction because folding both the **M** point and one **X** point to **Γ** results in folding of the other **X** point to **Γ** as well (Figure 1b). Figure S8 shows an experimental demonstration of a *p*2*mg* quadromer dimerized in the *x*-direction and along the along 45° diagonal that supports a pair of copolarized q-BICs.

**Figure 4:**
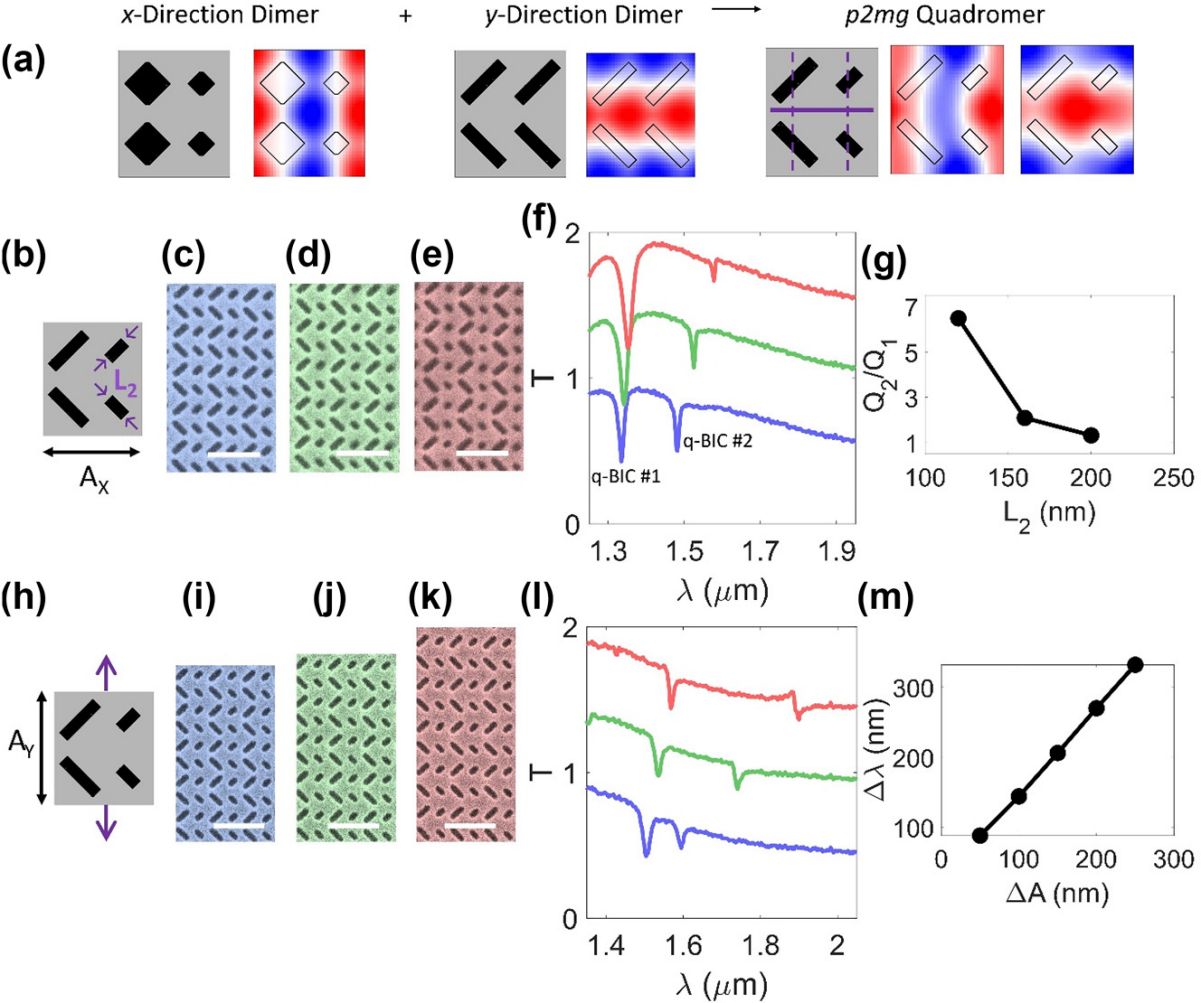
Experimental demonstration of engineering the Q-factors and spectral separation. (a) Construction of *p*2*mg* quadromer (with mirrors and glides marked with solid and dashed lines, respectively) supporting a pair of A q-BICs from *x*- and *y*-direction dimers. (b–g) Differentiation of Q-factors within pair of q-BICs. (a) Schematic of meta-unit. (c–e) False color scanning electron micrographs with decreasing L_2_. Scale bars: 1 µm. (f) Measured transmission spectra of devices in (c–e) for *y*-polarized incident light. Each spectrum offset by 0.5. (g) Calculated ratio of Q-factors (Q_2_/Q_1_ of q-BICs #2 and #1 respectively). (h–m) Spectral separation of modes by expanding lattice along *y*-direction. (h) Schematic of meta-unit. (i–k) False color scanning electron micrographs with increasing A_Y_. Scale bars: 1 µm. (l) Measured transmission spectra of devices in (i–k) for y-polarized incident light. Each spectrum offset by 0.5. (m) Measured separation between q-BICs as a function of lattice expansion along *y*-direction. Device dimensions detailed in Table S1.

Next, we experimentally demonstrate systematic control over the optical lifetimes and the spectral mode separation in a copolarized pair of q-BICs. We construct a *p*2*mg* quadromer supporting a pair of copolarized A q-BICs by combining a *p*2*mm* lattice dimerized in the *x*-direction with a *p*2*mg* lattice dimerized in the *y*-direction (Figure 4a). In typical pairs of degenerate or nearly degenerate q-BICs, both modes become radiative q-BICs under the same symmetry perturbation and thus have the same Q-factor. As such, monomer and dimer lattices do not support pairs of q-BICs where each q-BIC has a different Q-factor [6], [25]. Nonpaired q-BICs supported by different symmetry perturbations have Q-factors controlled by the strength of their respective perturbations, but they necessarily have unrelated mode profiles and poorly controlled frequency separation [6], [26]. In our quadromers (Figure 2d and e), each mode in the pair becomes a radiative q-BIC under a distinct symmetry perturbation. For the metasurface in Figure 4, the blueshifted mode of the pair is controlled by the size perturbation in the *x*-direction (q-BIC #1) and the redshifted mode by the perturbation along the *y*-direction to rotated rectangles (q-BIC #2). As such, we experimentally demonstrate that the two q-BICs can be engineered to have distinct Q-factors (Figure 4b–g). We fabricate a series of metasurfaces where the length of the smaller apertures is progressively shortened by 40 nm (Figure 4b–e). Shortening the apertures without changing their width increases the strength of the size perturbation but also decreases the strength of the rotated rectangle perturbation by making the rectangles more square-like. The measured spectra (Figure 4f) show a monotonic increase of the Q-factor of q-BIC #1 from ∼102 to ∼330 (due to the decreased in-plane aspect ratio of the apertures) and a slight decrease in the Q-factor of q-BIC #2 from ∼78 to ∼51 (from the increased size perturbation). The ratio of measured Q-factors of q-BIC #2 to q-BIC #1 reaches 6.48 for the device in Figure 4e (Figure 4g). We also demonstrate that the copolarized q-BICs can be spectrally separated by stretching or compressing the lattice along one axis (Figure 4h–m), which is largely independent of the geometrical parameters that control the Q-factors. We also fabricated a series of devices in which the lattice constant in the *y*-direction progressively increases (Figure 4h–j). As the *y*-direction perturbation is responsible for the redshifted q-BIC (q-BIC #2), increasing the *y*-direction lattice constant primarily redshifts q-BIC #2 and increases the mode separation (Figure 4l and m). The measured spectra show a mode separation of ∼332 nm for *x*- and *y*-direction lattice constants of 675 nm and 925 nm, respectively. Spectrally separating q-BICs by controlling the difference in *x*- and *y*-direction lattice constants avoids the reliance on fine-tuning precise nanostructure dimensions and enables a larger spectral separation than is typical among q-BICs [25], [28], [32]. Additionally, the selected q-BICs mode need not be surrounded by extraneous or higher-order modes, as is common in multiresonant metasurfaces [27], [28], [31], [34]. As an additional degree-of-freedom, the q-BICs in this lattice could be designed as cross-polarized instead of copolarized by rotating the polarization of the *y*-dimerized q-BIC by 90° via rotating the apertures by 45° to form a *p*2*mm* quadromer (Fig. S9). The examples in Fig. 4 and S9 together imply that the free-space polarization of the *y*-dimerized q-BIC is tunable to any intermediate polarization by appropriate rotation of the apertures [26], resulting in a pair of q-BICs with any desired combination of free-space polarizations.

**Figure 5:**
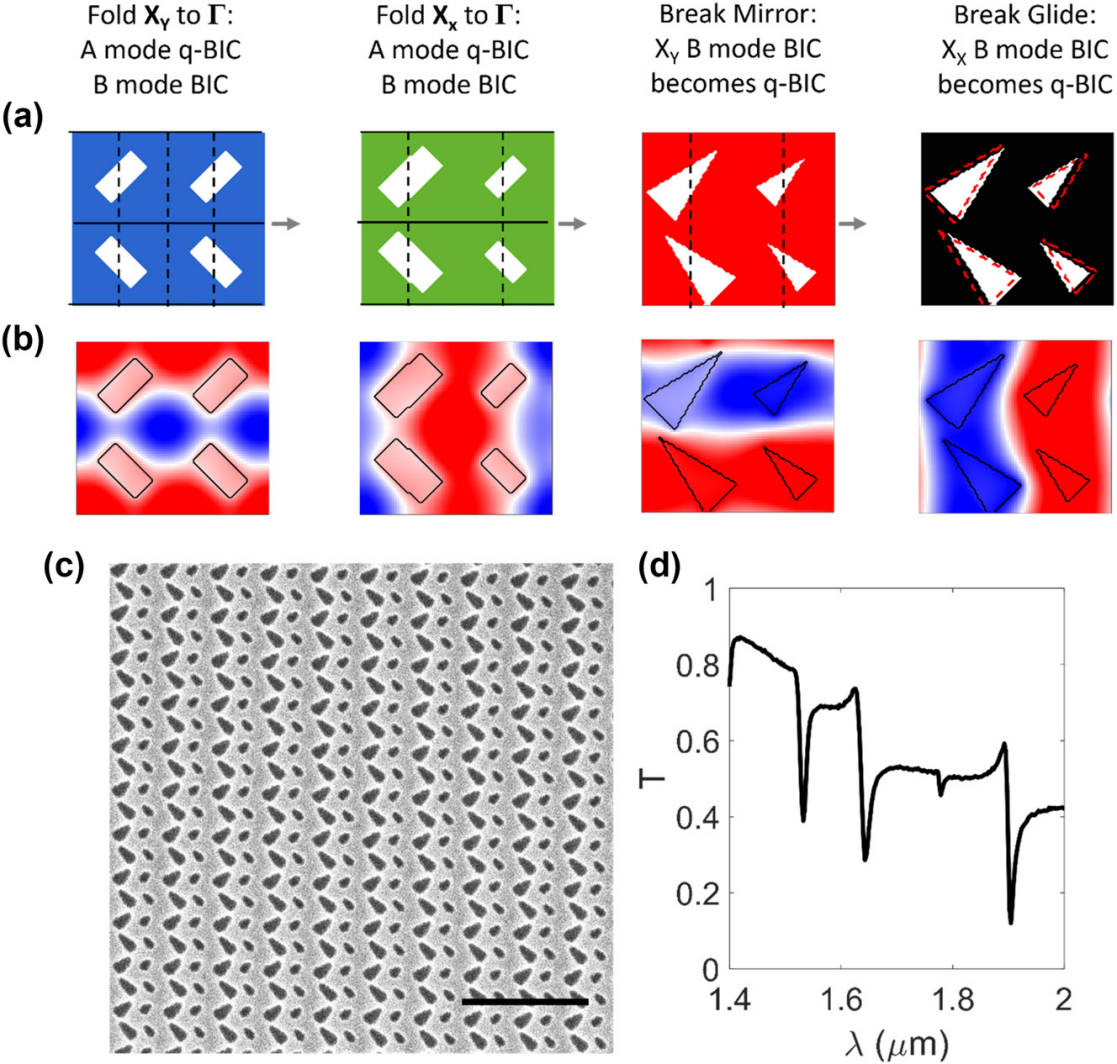
Experimental demonstration of copolarized quadruply resonant nonlocal metasurface. (a and b) Construction of quadruply resonant metasurface. (a) Schematic of successive symmetry perturbations. Mirrors and glides are marked with solid and dashed lines, respectively. Dashed trapezoids in leftmost panel indicate position of trapezoids prior to introducing spacing perturbation that breaks the glide symmetry. (b) Field profile of q-BIC introduced by each perturbation in (a). (c) Scanning electron micrograph of fabricated device (scale bar: 2 µm). Device dimensions detailed in Fig. S11. (d) Measured transmission for *y*-polarized incident light.

Taken together, the demonstrations of multiple nonlocal metasurfaces supporting distinct copolarized q-BICs suggest the possibility of quadruply resonant copolarized metasurfaces. In general, quadruply resonant metasurfaces from quadromer lattices can be formed by combining an *x*-dimerized and *y*-dimerized lattice where each dimer lattice supports both A and B q-BICs. Quadruply resonant metasurfaces with four copolarized q-BICs require judiciously chosen perturbations, as outlined in Figure 5a and b. In short, we combine the two quadromer lattices in Figures 3 and 4, as illustrated in Fig. S10, which is consistent with the principle that distinct perturbations support independent q-BICs. As a detailed explanation, we start with a *y*-direction dimerized *p*2*mg* lattice with a rotated rectangle perturbation. In this lattice, the A and B modes from **X**_**Y**_ have been folded to **Γ** such that the A mode is an *x*-polarized q-BIC and the B mode is a BIC. We then apply a dimerizing size perturbation in the *x*-direction, which folds A and B modes from **X**_**X**_ to **Γ** resulting in an *x*-polarized A q-BIC and a B BIC. The resulting lattice is a *p*2*mg* quadromer supporting A q-BICs and B BICs (Fig. S10). The metasurface can become quadruply resonant if additional symmetry perturbations convert the B BICs into radiative q-BICs. We first turn the *y*-dimerized B BIC into a q-BIC by changing the rectangles into triangles in a particular orientation, which breaks the mirror and 2-fold rotational symmetry but maintains the vertical glide. Introducing a spacing perturbation in the *x*-direction breaks the vertical glide and converts the *x*-dimerized B BIC into a q-BIC. All four q-BICs have the same dominant field component (H_Z_) and mode profiles that are shifted and/or rotated relative to each other (Fig. 5b and S11).

Finally, we experimentally demonstrate quadromer lattices that support four copolarized q-BICs. Quadruply resonant metasurfaces have been proposed but not experimentally demonstrated [26] or experimentally demonstrated with extra unwanted resonances, low Q-factors (Q<100), and poor fidelity [27] due to the severe nanofabrication constraints inherent in previous design methods. We again fabricate nonlocal metasurfaces as apertures etched into a thin film of silicon on a glass substrate (Figure 5c), and the measured spectrum shows four copolarized q-BICs (Fig. 5d and S11). The measured Q-factors range from Q∼140 to Q∼750 (Table S2) and show reasonable agreement with simulation considering spectrometer resolution limitations, minor but inevitable nanofabrication imperfections, and the angular dispersion of the q-BICs under measurement by a focused beam (Fig. S12). There are no unwanted modes in the spectrum, only deliberately chosen ones. As with the examples in Figure 4, stretching or compressing a lattice along one direction spectrally separates the *x*- and *y*-dimerized modes (Fig. S13). We have highlighted copolarized quadruply resonant metasurfaces for their improbability. However, quadromer lattices offer many options for multiresonant nonlocal metasurfaces. Triply resonant metasurfaces are realized by excluding one perturbation, and quadruply resonant metasurfaces where selected q-BICs are cross-polarized are achieved by choosing a different combination of perturbations (Fig. S14).

## 3 Conclusions

Although monomer and dimer lattices with broken symmetry have been studied exhaustively, expanding beyond dimer lattices immediately introduces new options for optical capabilities. We experimentally demonstrated nonlocal quadromer metasurfaces that support pairs of q-BICs with free-space polarizations, Q-factors, spectral separations, and mode profiles that are readily selectable through a symmetry-based rational design paradigm. We then further extended our design paradigm to experimentally demonstrate quadruply resonant metasurfaces whose design enables well-controlled q-BICs without imposing stringent nanofabrication constraints. Our design scheme narrows the design space of multiresonant nonlocal metasurfaces into a tractable problem with only a few key parameters that control different q-BIC properties in independent and predictable ways – lattice symmetry determines polarizations and mode profiles of multiple resonances, the *x*- and *y*-direction lattice constants control the frequency spacing between modes, and the perturbation strength of each q-BIC influences its Q-factor. Additionally, we can select any motif that is compatible with the prescribed lattice symmetry, which allows simple-to-fabricate nanostructures such as rectangles, trapezoids, and triangles. Compared to the results in Figures 3–5, recent multiresonant metasurfaces designed by inverse design techniques [21], [27], [37], [38], [39] require more expensive computation and fabrication, sometimes only to yield less comprehensive design control (e.g., no choice of field profile [38], [39]) or worse optical performance (such as very low experimental Q-factors that are more than an order of magnitude worse than their design [27]). Rational design of more-than-dimer lattices offers a promising pathway for developing advanced q-BIC capabilities, which should encourage further exploration of alternative lattices [6] such as hexagonal quadromer lattices.

As our design paradigm is rooted in lattice symmetry rather than idiosyncrasies of the nanostructure dimensions or their material properties, it is readily generalizable to other dielectric materials and wavelength ranges and, therefore, may enable a wide variety of applications. For example, the capability to spectrally separate q-BICs across a large tuning range will prove particularly useful for applications in nonlinear optics requiring resonances at prescribed frequencies. Additionally, the ability to select pairs of either A or B q-BICs in quadromer lattices enables improved flexibility relative to monomer and dimer lattices for designing multiresonant metasurfaces with optimal field overlap with an active material for specific applications (i.e., field concentrated in the gaps between nanostructures for multicolor optical sensors [32] but in the metasurface for multiresonant modulators [10]). Engineerable and fabricable quadruply resonant metasurfaces represent an unprecedented capability for nonlocal metasurfaces and can dramatically improve metasurface performance in applications that require multiple optical resonances such as quantum and nonlinear optics, multicolor augmented reality headsets, and multicolor spectral filters. Meta-optics with even more resonances can be achieved by cascading [24] or spatially multiplexing [29] distinct quadruply resonant metasurfaces.

## 4 Methods

Sample fabrication: All devices were fabricated on a 200 nm polycrystalline silicon thin film on JGS2 fused silica substrate. The samples were spincoated with ZEP520A resist and an anticharging layer (DisChem Inc., DisCharge), patterned by a 100 keV electron-beam lithography system, and developed in n-amyl acetate. The silicon thin film was etched by a reactive ion etch process with SF_6_ and C_4_F_8_. The remaining ZEP resist was removed by PG remover at 80 °C.

Electromagnetic Simulations: Metasurfaces were designed by finite-difference time domain in Lumerical FDTD with periodic boundary conditions. Bandstructures were calculated by guided mode expansion in Legume [40], [41].

## Supplementary Material

Supplementary Material Details
