# Supplementary material for: Robust multiresonant nonlocal metasurfaces by rational design
Source: Nanophotonics. 2025 Feb 4;14(4):449–58. doi: 10.1515/nanoph-2024-0551 (PMC11834050; doi:10.1515/nanoph-2024-0551)
Supplement: Supplementary file 1 — Supplementary Material Details [file j_nanoph-2024-0551_suppl_001.pdf]

Supplementary information for

Robust Multi-Resonant Nonlocal Metasurfaces by Rational Design

Stephanie C. Malek[1*], Chloe F. Doiron[1], Igal Brener[1], and Alexander Cerjan[1*]

[1]Center for Integrated Nanotechnologies, Sandia National Laboratories, Albuquerque, NM, 87185, USA

*email: scmalek@sandia.gov, awcerja@sandia.gov

Supplementary information Section 1: Intuitive Construction of Cross-Polarized Pairs of q-BICs in Quadromer Lattices

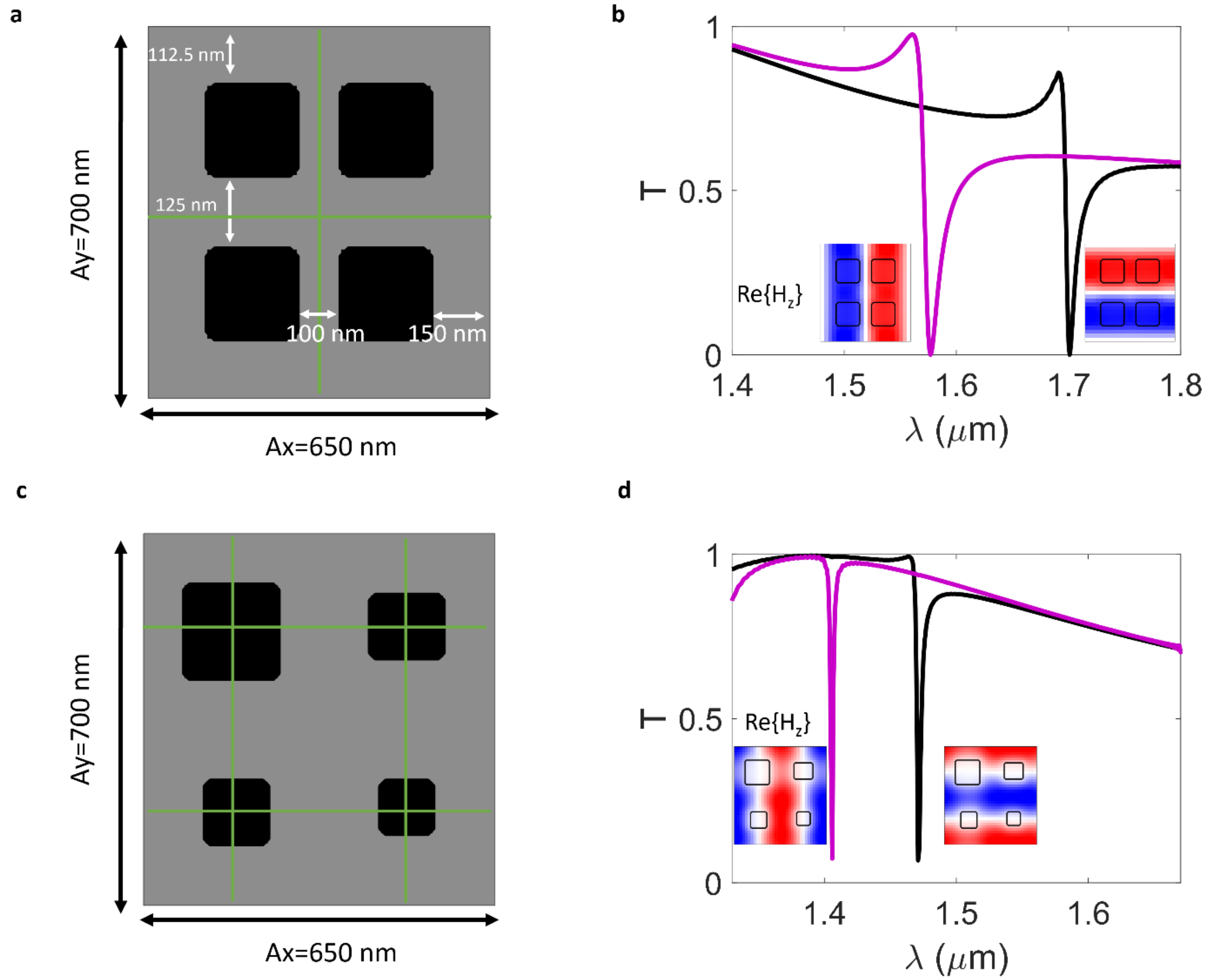

Figure S1: Metasurfaces with *p2mm* quadromer lattices supporting cross-polarized pairs of q-BICs by the introducing same dimerizing perturbation in the *x*- and *y*-directions. (a-b) Metasurfaces supporting B q-BICs with dimerizing *spacing* perturbation in *x*- and y-directions. (a) Schematic of quadromer meta-unit with mirror symmetry operations marked in green. (b) Simulated transmission spectra for *x*-polarized (black) and *y*-polarized (purple) light. Insets: Out-of-plane component of magnetic field of q-BICs. (c-d) Metasurfaces supporting A q-BICs with dimerizing *size* perturbation in *x*- and *y*-directions. (c) Schematic of quadromer meta-unit with mirror symmetry operations marked in green. (d) Simulated transmission spectra for *x*-polarized (black) and *y*-polarized (purple) light. Insets: Out-of-plane component of magnetic field of q-BICs.

Supplementary information Section 2: Theory

'Selection rules' detail which modes are BICs vs. q-BICs in a given structure and which free-space polarization couples to each q-BIC. We derive selection rules for relevant quadromer lattices following the process outlined in [1]. Three key points from [1] are relevant to the construction of quadromer lattices:

1. Within a given lattice, selection rules are derived by separating perturbations of different periodicities and considering their selection rules separately. Additionally, symmetry perturbations with different periodicity support different sets of modes. As such, we treat *x*- and *y*-dimerized modes and symmetry perturbations separately in quadromer lattices.
2. Selection rules can be derived in several equivalent ways (i.e., graphically or through calculation of a coupling integral), though Group Theory is often the most efficient method.
3. To determine if a mode is a BIC or a q-BIC, we consider the free-space coupling condition, which for a perturbed nonlocal metasurface is reduced to:

$$\Gamma_{\text{Free-Space}} = \Gamma_{\text{V}} \otimes \Gamma_{\Psi}$$

where Γ is an irreducible representation (irrep) and $\otimes$ is a direct product representation. Therefore, Γ_{V} denotes the irreducible representation of the perturbed structure, Γ_{Ψ} is the irreducible representation of the mode, and $\Gamma_{\text{free-space}}$ is the irreducible representation of free-space polarized light. Physically, the equation means that if perturbed portion of the mode ($\Gamma_{\text{V}} \otimes \Gamma_{\Psi}$) has a non-zero net dipole moment, it can couple to free space at normal incidence (i.e., be a q-BIC rather than a BIC). The direction of its net dipole moment is the direction of the free-space polarization that can excite the q-BIC.

To derive selection rules of quadromer lattices, we must determine the irreducible representation of (1) the perturbed lattices, (2) the relevant modes, and (3) the free-space polarization. The irreducible representations can all be deduced by reference to the appropriate character table (**Fig S1a**) in conjunction with examining the symmetry. Determinations of relevant irreducible representations are shown in **Fig S1c-e**.

We explicitly derive selection rules for the two cases of co-polarized pairs of q-BICs described in **Fig. 2d-e** of the Main Text (**Fig. S2**). We consider *p2mg* quadromers as the sum of particular *x*-dimerized and *y*-dimerized lattices. **Fig. S1d** outlines the irrep of constituent *x*-dimerized and *y*-dimerized lattices, and **Fig S1e** shows irreps of the relevant modes. For each quadromer, we separate the *x*- and *y*-dimerized portions and take the direct product representation with the relevant modes (**Fig S1b** or by computation from character table in **Fig. S1a**). If the resulting direct product representation is the same as a possible free-space polarization (i.e., B_1 or B_2 from **Fig. S1c**), the lattice supports a q-BIC excited by the indicated free-space polarization. The same process can be followed to derive selection rules for *p2mm* or *p2gg* quadromer lattices with cross-polarized q-BICs, or *p2mm*, *p2mg*, and *p2gg* lattices with mismatched q-BICs from different bands (i.e., an A and B mode rather than pair of A or B modes).

a

	E	C_2	σ_X	σ_y
**A_1**	1	1	1	1
**A_2**	1	1	-1	-1
**B_1**	1	-1	1	-1
**B_2**	1	-1	-1	1

b

	**A_1**	**A_2**	**B_1**	**B_2**
**A_1**	A_1	A_2	B_1	B_2
**A_2**	A_2	A_1	B_2	B_1
**B_1**	B_1	B_2	A_1	A_2
**B_2**	B_2	B_1	A_2	A_1

c

	**TM modes**	**TE modes**
**B_1**	X	Y
**B_2**	Y	X

d

	p2mm	***p2mg***	***p2mm***	***p2mg***
**X-Direction Dimerized Unit Cell (V_X)**	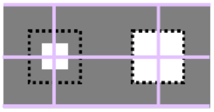 Size Perturbation	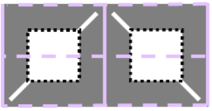	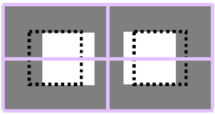 Spacing Perturbation	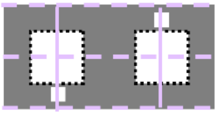
**Irreducible Representation of Perturbed Portion (Γ_{VX})**	B_1	B_2	A_1	A_2
**Y-Direction Dimerized Unit Cell (V_Y)**	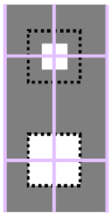	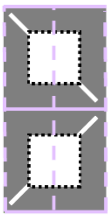	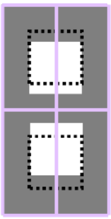	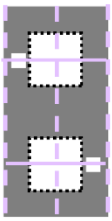
**Irreducible Representation of Perturbed Portion (Γ_{VY})**	B_2	B_1	A_1	A_2

e

	X-Dimerized A mode	Y-Dimerized A mode	X-Dimerized B Mode	Y-Dimerized B Mode
**Modes of Interest (Ψ)**	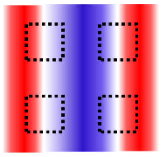	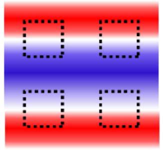	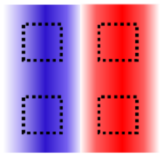	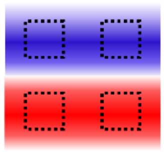
**Irreducible Representation (Γ_Ψ)**	A_1	A_1	B_1	B_2

Figure S2: Group Theory irreducible representations. (a) Character table for mm2 point group. C_2 is a 2-fold rotation and σ denotes a mirror. A value of 1 in the character table indicates symmetry about the relevant symmetry operation and a value of -1 denotes anti-symmetry. (b) Direct product representation of relevant irreducible representations. (c) Irreducible representations of free-space polarizations. (d) Irreducible representations of relevant perturbed lattices. (e) Irreducible representations of relevant modes.

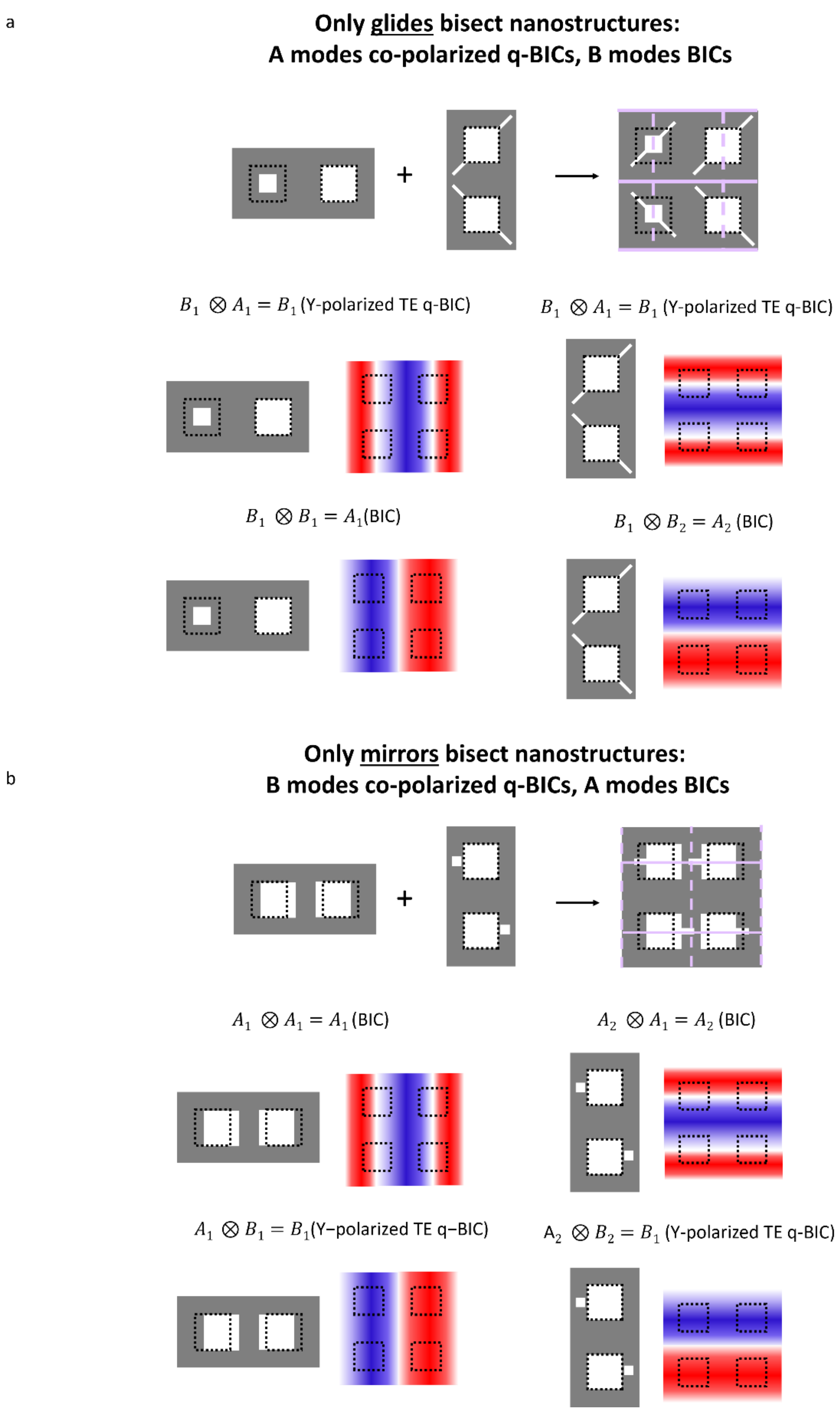

Figure S3: Group Theory derivation of selection rules in selected *p2mg* quadromer lattices supporting pairs of (a) A q-BICs and (b) B q-BICs.

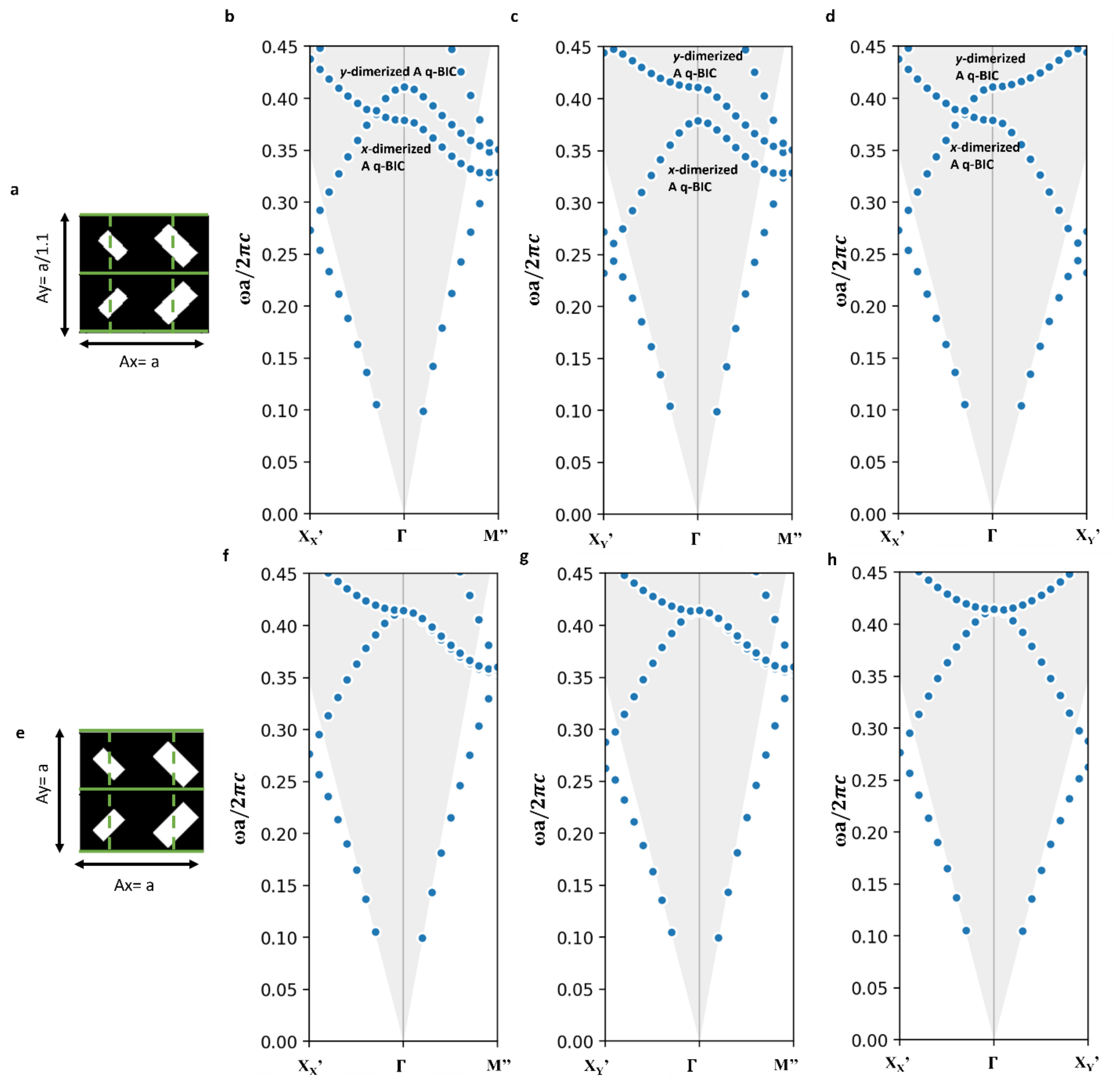

Figure S4: Representative photonic bandstructure of pair of TM A q-BICs. (a) Meta-unit schematic for *rectangular* meta-unit with non-degenerate bands. (b-d) Calculated bandstructures along different directions of the quadromer FBZ. (e) Meta-unit schematic for *square* meta-unit. (f-h) Calculated bandstructures along different directions of the quadromer FBZ.

Supplementary information Section 3: Additional Experiments and Simulations for Co-Polarized q-BICs

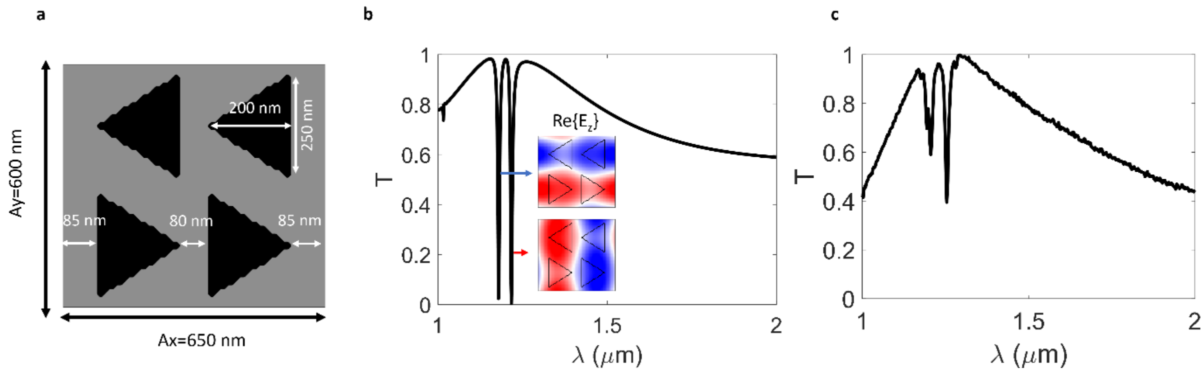

Figure S5: Additional information for co-polarized B q-BICs in **Fig. 3** of Main Text. (a) Schematic of quadromer meta-unit. (b-c) Simulated (b) and measured (c) transmission spectra for *x*-polarized incident light. (b) Insets: Mode profiles of TM q-BICs.

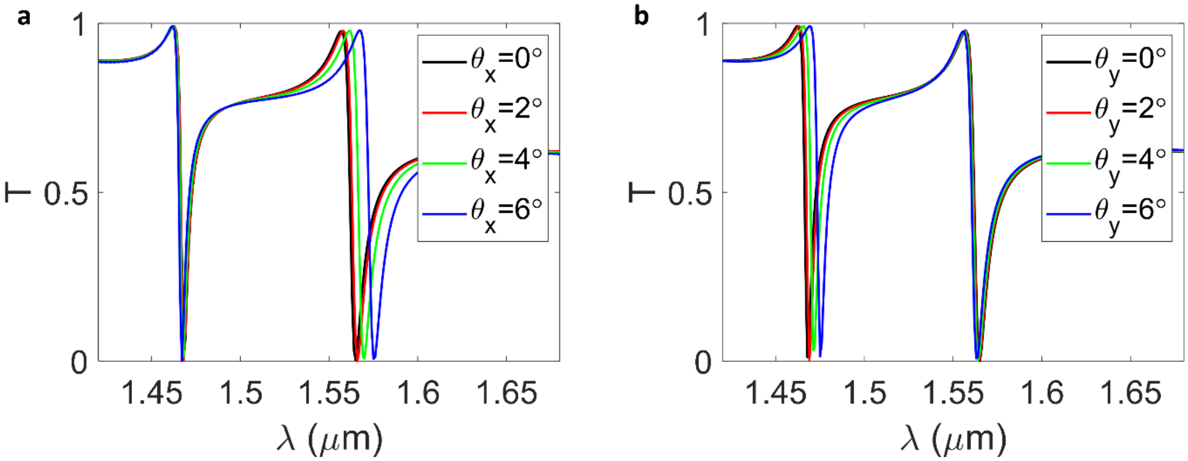

Figure S6: Dependence of resonant wavelength on incident angle for TE B q-BICs in metasurface in **Fig. 3** of the Main Text. (a-b) Simulated transmission spectra at off-normal incidence in the *x*-direction (a) and *y*- direction (b).

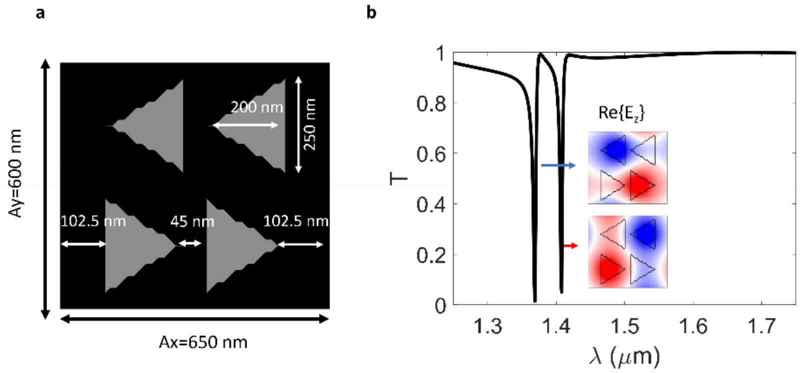

Figure S7: Metasurface of 1 μm tall pillars supporting co-polarized B q-BICs (a) Schematic of quadromer meta-unit. (b) Simulated transmission spectra for *x*-polarized incident light. (b) Insets: Mode profiles of TM q-BICs.

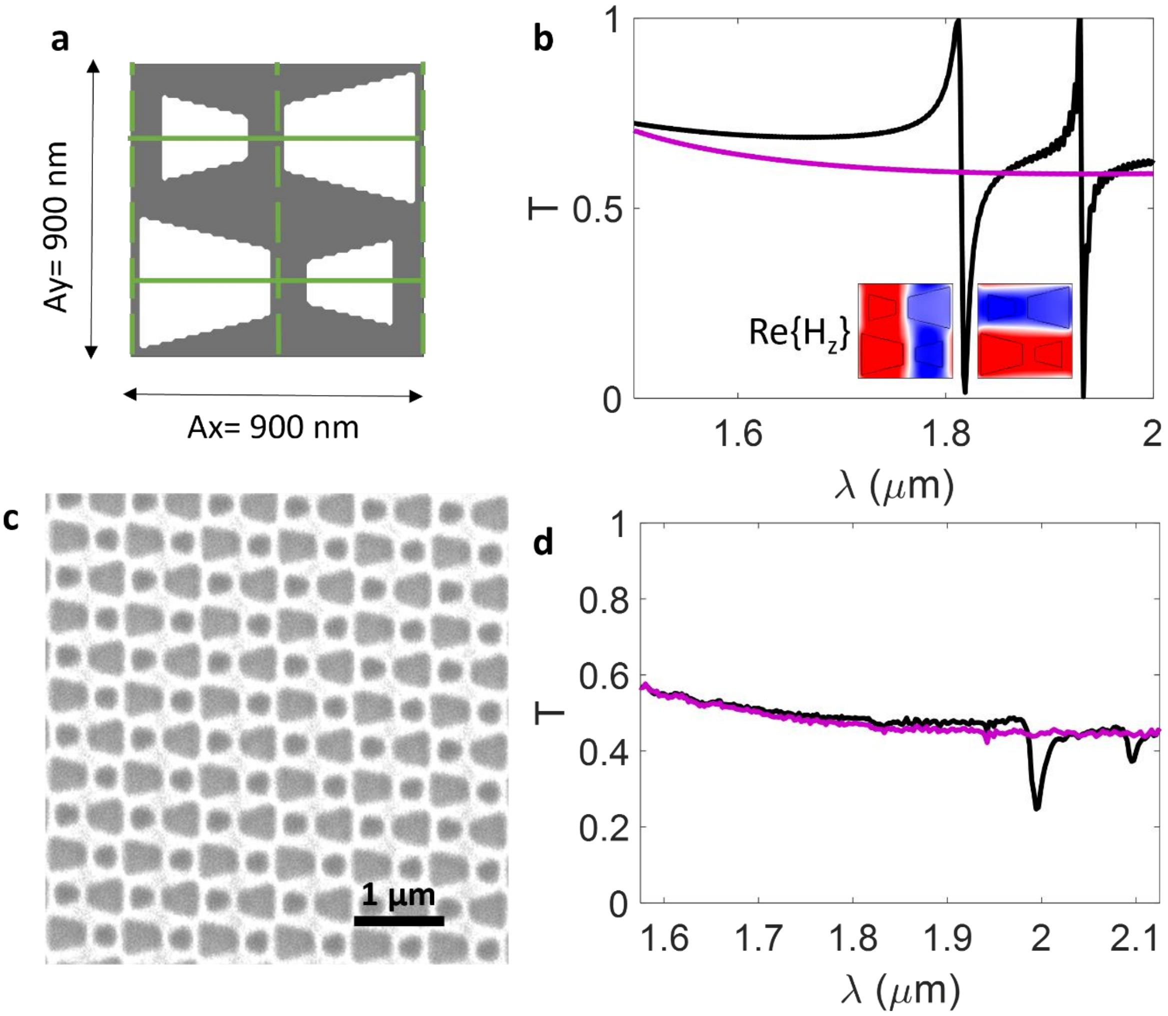

Figure S8: Experimental demonstration of co-polarized pairs of q-BICs from dimerizations in *x*-direction and diagonal direction. (a) Schematic of *p2mg* quadromer meta-unit with mirror and glide symmetry operations marked in green. (b) Simulated spectra for *y*-polarized (black) and *x*-polarized (purple) incident light. Insets: Out-of-plane magnetic field of q-BICs. (c) Scanning electron micrograph of fabricated device. (d) Measured transmission spectra for *y*-polarized (black) and *x*-polarized (purple) incident light.

	Ax (nm)	Ay (nm)	Rectangle 1 (nm)	Rectangle 2 (nm)
Fig. 4c-e	675	775	320 x 80	200, 160, 120 x 80
Fig. 4h-j	675	725, 825, 925	320 x 80	200 x 80

Table S1: Designed dimensions for devices in **Fig. 4** of Main Text.

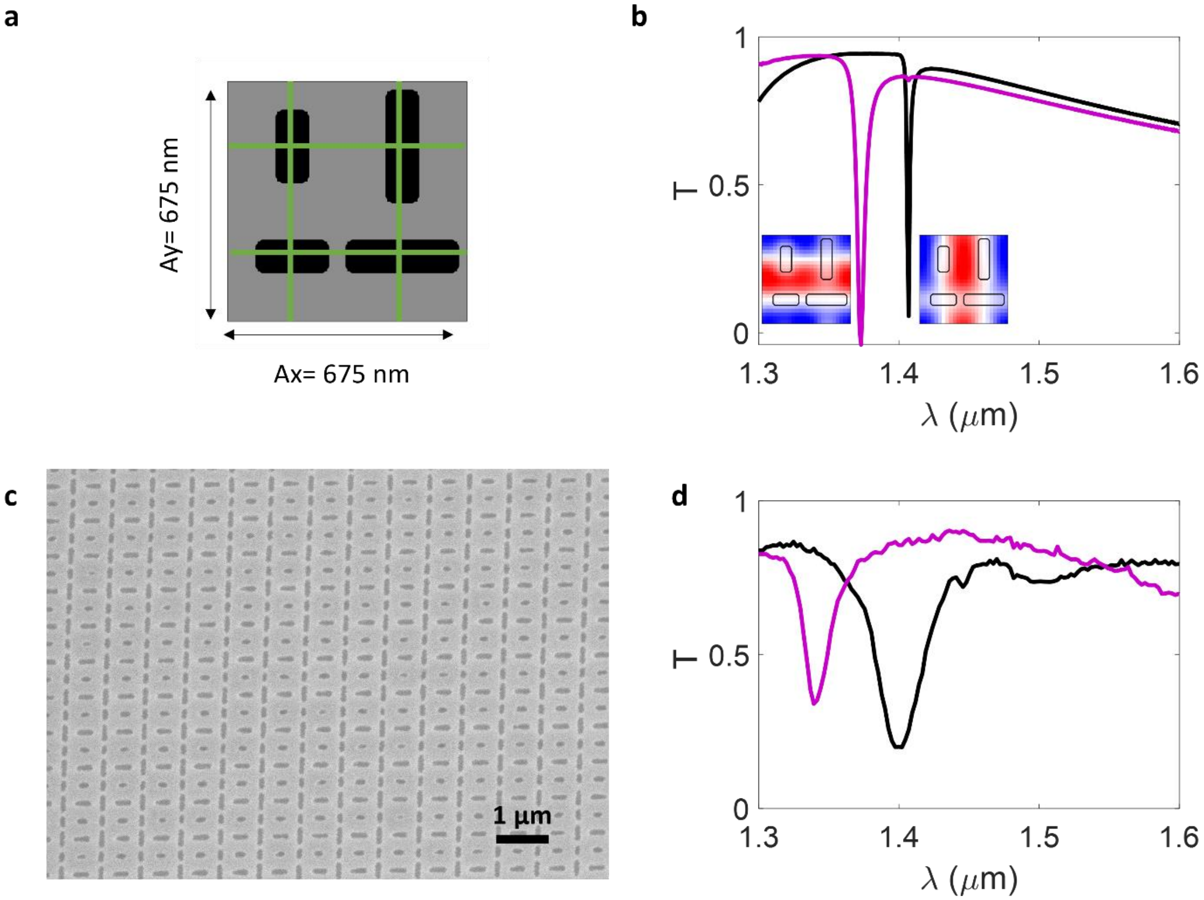

Figure S9: Experimental demonstration of metasurface with *p2mm* quadromer lattice supporting cross-polarized pairs of A q-BICs. (a) Schematic of quadromer meta-unit with mirror symmetry operations marked in green. (b) Simulated spectra for *y*-polarized (black) and *x*-polarized (purple) incident light. Insets: Out-of-plane component of magnetic field profiles of q-BICs. (c) Scanning electron micrograph of fabricated device. (d) Measured transmission spectra for *y*-polarized (black) and *x*-polarized (purple) incident light. Unlike the devices in the main text, the experimental Q-factors in this metasurface are lowered by nanofabrication issues associated with an insufficient and uneven anti-charging layer for lithography.

Supplementary Information Section 4: Quadruply Resonant Metasurfaces

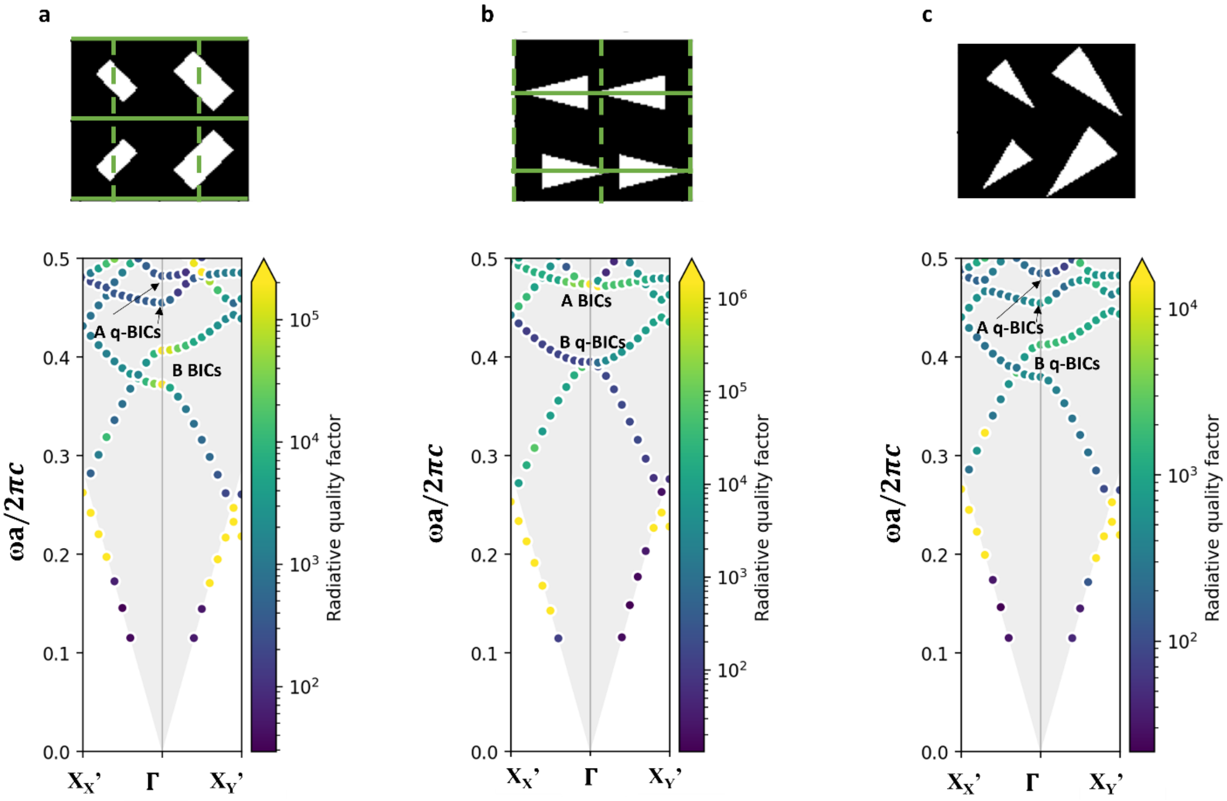

Figure S10: Construction of quadruply resonant metasurface from quadromers supporting co-polarized pairs of q-BICs. (a) Bandstructure of *p2mg* quadromer (with glides (dashed lines) intersecting the nanostructures) supporting co-polarized pairs of A q-BICs and B BICs. (b) Bandstructure of *p2mg* quadromer with mirrors (solid lines) intersecting nanostructures supporting co-polarized pairs of B q-BICs and A BICs. (b) Bandstructure of quadromer with no mirrors or glides supporting pairs of co-polarized A and B q-BICs. Material fill-fraction is held constant in (a-c).

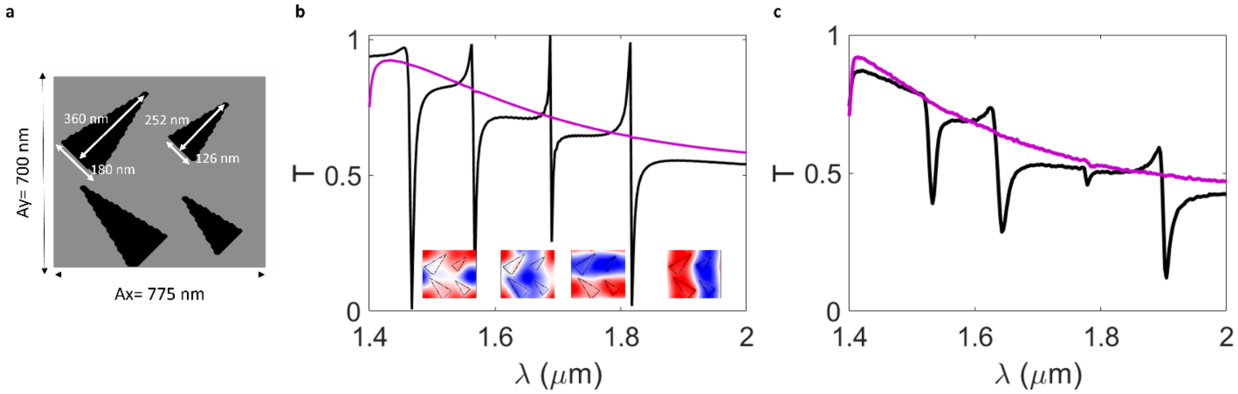

Figure S11: Additional information for co-polarized quadruply resonant metasurface in **Fig. 5** of Main Text. (a) Schematic of meta-unit. The spacing perturbation in the *x*-direction pushes every other nanostructure 50 nm closer to the neighboring nanostructure. (b) Simulated transmission spectra for *y*-polarized (black) and *x*-polarized (purple) incident light. Insets: Out-of-plane component of magnetic field of q-BICs. (c) Measured transmission spectra for *y*-polarized (black) and *x*-polarized (purple) incident light.

**Mode**	**Simulated (FDTD)**	**Measured**
y-dimerized A q-BIC	233	160
x-dimerized A q-BIC	467	139
y-dimerized B q-BIC	1302	749
x-dimerized B q-BIC	585	269

Table S2: Q-factors for quadruply resonant metasurface in **Fig. 5** of the main text.

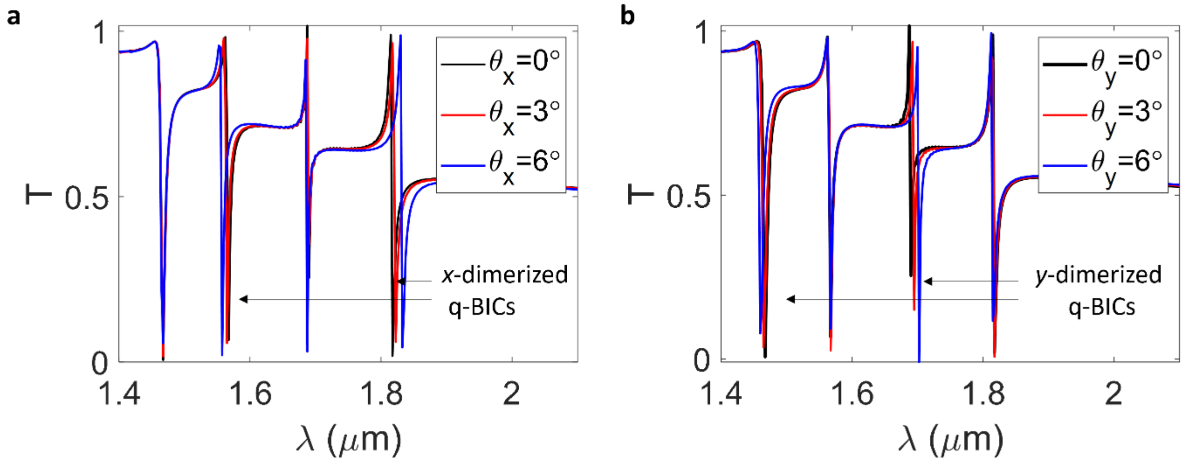

Figure S12: Dependence of resonant wavelength on incident for angle quadruply resonant metasurface in **Fig. 5** of the Main Text. (a-b) Simulated transmission spectra at off-normal incidence in the *x*-direction (a) and *y*- direction (b).

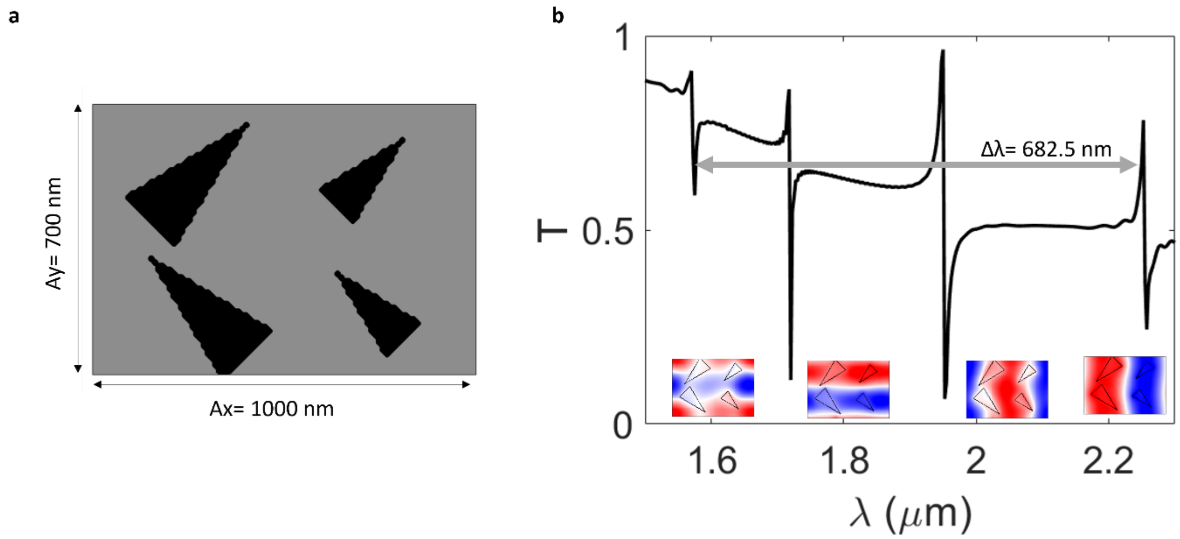

Figure S13: Simulated control of mode separation of quadruply resonant metasurface in **Fig. 5** of Main Text. (a) Meta-unit schematic. (b) Simulated transmission spectra for *y*-polarized incident light. Insets: Out-of-plane component of magnetic field of q-BICs. Note that significantly increasing Ax separates modes spectrally and also rearranges order of modes relative to **Fig. 5** of Main Text (i.e., *x*-dimerized A and *y*-dimerized B q-BICs switched compared to **Fig. 5**).

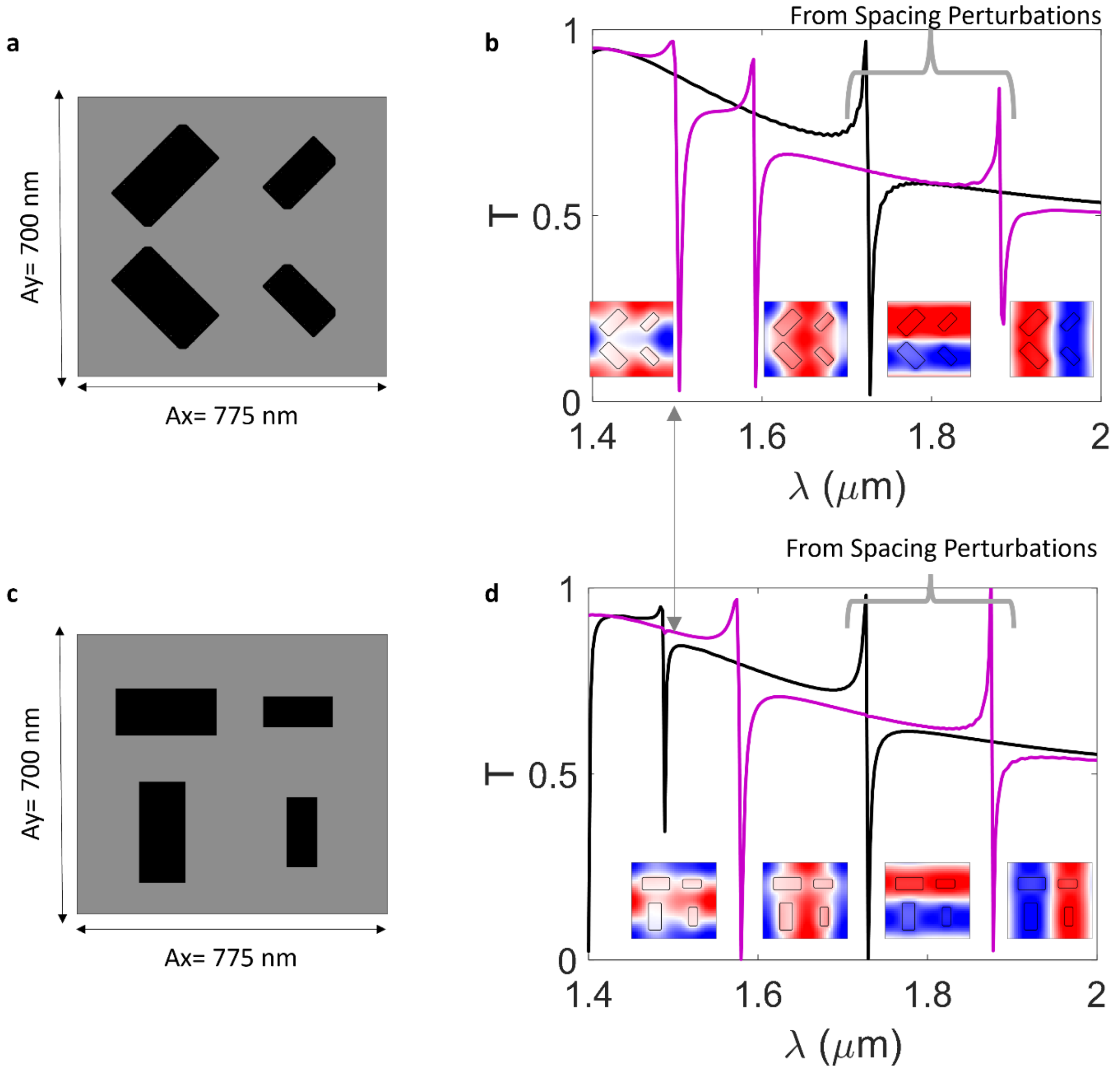

Figure S14: Examples of quadruply resonant metasurfaces with selected q-BICs cross-polarized. (a-b) Metasurface with co-polarized A q-BICs and cross-polarized B q-BICs (A q-BICs supported by same perturbations as device in Fig. 4 of main text and B q-BICs supported by additional spacing perturbations in *x*- and *y*-directions). (a) Schematic of meta-unit. The spacing perturbation is 50 nm. (b) Simulated transmission spectra for *x*-polarized (black) and *y*-polarized (purple) incident light. Insets: Out-of-plane component of magnetic field of q-BICs. (c-d) Metasurface with pairs of cross-polarized A and B q-BICs. A q-BICs become cross-polarized due to nanostructure rotation relative to (a), as in Fig. S6. (c) Schematic of meta-unit. The spacing perturbation is 50 nm. (d) Simulated transmission spectra for *x*-polarized (black) and *y*-polarized (purple) incident light. Insets: Out-of-plane component of magnetic field of q-BICs.

References

(1) Overvig, A. C.; Malek, S. C.; Carter, M. J.; Shrestha, S.; Yu, N. Selection Rules for Quasibound States in the Continuum. *Phys. Rev. B* **2020**, *102* (3), 035434. https://doi.org/10.1103/PhysRevB.102.035434.
